# Phytotoxic Effects of the Aqueous Extracts of *Magnolia biondii* Pamp. Flower Litter and the Joint Action of Allelochemicals

**DOI:** 10.3390/plants14111577

**Published:** 2025-05-22

**Authors:** Yi Yu, Yalei Du, Jiajia Dong, Zhigang Yin, Peiyu Chen, Lingling Cao, Zhiqiang Yan

**Affiliations:** 1 School of Life Sciences, Nanyang Normal University, Nanyang 473061, China; yuyi992594@163.com (Y.Y.); dyl2024315@163.com (Y.D.); dong20231120@163.com (J.D.); 2 Nanyang Academy of Sciences, Nanyang 473000, China; gangzhiyin@163.com (Z.Y.); chenpeiyu1984@163.com (P.C.); 3 College of Water Resources and Modern Agriculture, Nanyang Normal University, Nanyang 473061, China; 4 Collaborative Innovation Center of Water Security for Water Source Region of Mid-Line of South-to-North Diversion Project of Henan Province, Nanyang Normal University, Nanyang 473061, China

**Keywords:** *Magnolia biondii*, flower litter, phytotoxicity, allelochemical, joint action

## Abstract

*Magnolia biondii* Pamp., an elegant ornamental tree that graces early spring landscapes, has flower buds that are widely used as Traditional Chinese Medicine ‘Xin Yi’. In this study, the phytotoxic effects of aqueous extracts derived from tepal litter (EMT) and bract litter (EMB) of *M. biondii* flower were evaluated on six target plant species: *Triticum aestivum* L., *Lactuca sativa* L., *Zoysia pacifica*, *Agrostis canina* L., *Trifolium pratense* L. and *Axonopus compressus*. Secondary metabolites in EMT and EMB were tentatively characterized by liquid chromatography high-resolution mass spectrometry (LC-HRMS), and the joint action of allelochemicals was examined. Our findings revealed that both EMT and EMB significantly inhibited the seed germination and seedling growth of all target plants in a concentration-dependent manner. There were 120 and 98 secondary metabolites annotated by LC-HRMS in EMT and EMB, respectively. Among them, malic acid (MA) and citric acid (CA) with high relative contents showed synergistic phytotoxicities on seed germination and seedling growth of *L. sativa* and *Z. pacifica*. In summary, the flower litter of *M. biondii* displayed significant allelopathic effects, and the synergistic effects of MA and CA probably played an important role.

## 1. Introduction

*Magnolia biondii* Pamp. is a deciduous tree belonging to the Magnoliaceae family, primarily distributed in East, Central, and Southwest China [1]. With a cultivation history spanning more than 2500 years in China, *M. biondii* was introduced to Europe and the Americas, and has now become a cherished early spring flower beloved worldwide [2]. *M. biondii* exhibits a graceful form and exceptional ornamental value, frequently depicted in literary works across historical and global contexts, thereby embodying profound historical and cultural significance. *M. biondii* exhibits rapid growth rates, robust adaptability, and a wide geographical distribution, thus, it is extensively cultivated in diverse environments. Its vivid floral pigmentation and pronounced fragrance underpin its critical role in both forestry and horticultural industries. Additionally, the species’ versatility in landscape greening highlights its dual ecological functionality and aesthetic value, further solidifying its importance in sustainable ecosystem design [3,4].

Besides the ornamental value, *M. biondii* is also used as a Traditional Chinese Medicine. The dried flower buds are the medicinal parts named ‘Xin Yi’, growing annually from March to May. The medicine is listed in ‘China Pharmacopoeia Commission’, effectively promotes nasal orifice ventilation and heals wind-cold, treating symptoms such as runny nose, nasal congestion, and wind-cold headache [5,6]. Modern pharmacological studies indicated that the main active components of Xin Yi are primarily found in the essential oil fraction. Volatile compounds including ketones, aldehydes, alcohols, esters, phenols, ethers and terpenes were identified by GC-MS. Among them, terpenes and alcohols were revealed as the main types of flavor compounds, e.g., eucalyptol, β-pinene, linalool, α-terpineol, and sabinene [7,8]. The essential oil of Xin Yi exhibited antiviral, antioxidant, and anti-allergic activities. It can inhibit the release of inflammatory factors, alleviate nasal mucosal edema and allergic reactions, and demonstrate significant therapeutic effects on allergic rhinitis and chronic rhinitis [9]. Besides the essential oil, Xin Yi contains a lot of other secondary metabolites, such as flavonoid glycosides, alkaloids, and lignans. These compounds showed various biological activities, including antioxidant, antitumor, antibacterial, anti-allergic, anti-inflammatory, immunomodulatory, neuroprotective, and anti-hyperlipidemic effects [10,11,12,13,14,15,16,17,18,19,20].

Allelopathy, the ecological phenomenon whereby plants release secondary metabolites (allelochemicals) to exhibit stimulatory or inhibitory effects on neighboring plants, microorganisms, or their growth, serves as a critical driver of ecosystem dynamics [21]. In both natural and agricultural systems, allelopathy influences vegetation succession by favoring the dominance of specific species, thereby shaping local biodiversity [22]. Allelochemicals are plant secondary metabolites with phytotoxic activities and ecological functions. To date, 14 groups of allelochemicals have been identified from plants, primarily including phenolic compounds, alkaloids, terpenoids, glucosinolates and isothiocyanates, benzoxazinoids, and miscellaneous compounds [23]. Allelochemicals play a critical role in eco-friendly weed control while also serving as plant growth regulators and signaling molecules [24,25,26].

Plants release allelochemicals through pathways such as root exudation, volatilization from stems and leaves, leaching via rainwater, and decomposition of plant litter [27]. Among them, plant litter is one of the main sources of allelochemicals in agroforestry systems. The release of allelochemicals during litter decomposition introduces bioactive compounds into soil ecosystems, which induce alterations in soil physical and chemical properties and modify microbial community composition, structure, and diversity. These transformations profoundly alter the rhizosphere microecology, creating biochemical barriers that inhibit seed germination and seedling establishment in adjacent vegetation. Under persistent allelopathic pressure, such ecological modifications may ultimately trigger plant community degradation through progressive species displacement [28]. For instance, the invasive plant *Ageratina adenophora* (Crofton weed) releases flavonoids through plant litter decomposition, inhibiting the growth of native plants and exacerbating ecological invasion [29]. The aqueous extracts of *Paulownia tomentosa* flower litter showed strongly inhibitory effects on seed germination and seedling growth of wheat, lettuce, green bristlegrass, and purslane, indicating that *P. tomentosa* flower litter was an effective source to release allelochemicals [30]. Decomposing leaf litter is one of the main sources of allelochemicals in walnut agroforestry systems. Lettuce growth and physiological processes were inhibited by walnut leaf litter, especially during the early growth stage [31]. Therefore, plant litter was an important source of allelochemicals, which played key roles in the allelopathic interaction between plants and their surrounding organisms.

Previous studies revealed that several *Magnolia* plants exhibited allelopathic effects on target plants. The leaf litter extract of *M. grandiflora* inhibited the root and shoot growth of ryegrass in a concentration-dependent manner [32]. Lignans from the leaves of *M. liliflora* showed an inhibitory effect on lettuce shoots with an IC_50_ between 0.37 and 0.95 mmol/L [33]. Cyclocolorenone, a sesquiterpene ketone, was isolated from *M. grandiflora* by a bioassay-directed fractionation. It showed significant growth inhibitory activities in an etiolated wheat coleoptile assay, and was also phytotoxic to greenhouse-grown corn, bean, and tobacco [34]. Two sesquiterpene lactones, costunolide and parthenolide, were isolated from the leaves of *M. grandiflora*. Both sesquiterpenes showed pronounced inhibition of seed germination and root and shoot growth of four test plants: wheat, lettuce, radish, and onion. Interestingly, parthenolide reduced germination and inhibited seedling growth more than costunolide, and the inhibition of root growth was generally greater than that of shoot growth [35].

These studies indicated that plants in the genus *Magnolia* possess abundant secondary metabolites with significant allelopathic potential. This study focuses on *M. biondii*, aiming to: (1) evaluate the allelopathic effects of its flower litter on several crops and turfgrass plants, (2) analyze the composition of aqueous extracts of *M. biondii* flower litter, and (3) identify potential allelochemicals and evaluate their joint actions. The findings will clarify the allelopathic interactions between *M. biondii* and neighboring plants, providing a theoretical foundation for its rational allocation in horticultural and agricultural ecosystems. To our knowledge, this is the first report on the allelopathic potential of *M. biondii* flower litter.

## 2. Results

### 2.1. Effects of EMT and EMB on Seed Germination

**Figure 1:**
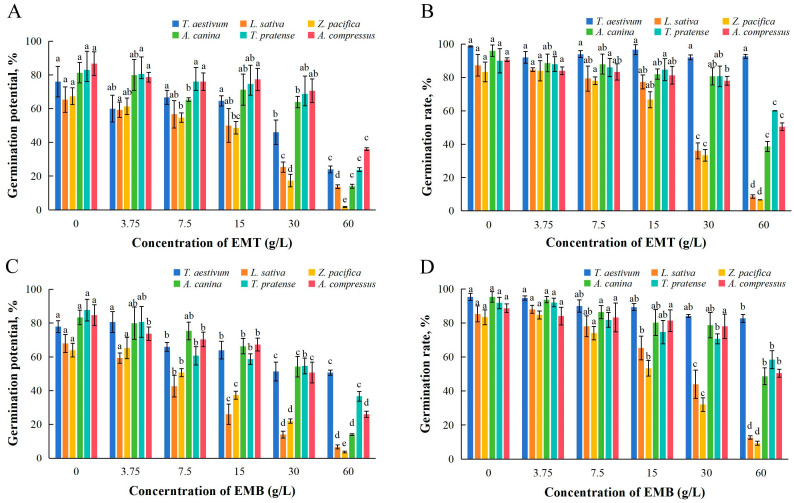
Germination potential (**A**,**C**) and germination rate (**B**,**D**) of six plant seeds under treatment with EMT (**A**,**B**) and EMB (**C**,**D**). The results presented are the mean of three replicates ± SE; different letters denote significant differences at *p* < 0.05 according to one-way ANOVA with an LSD test.

As shown in Figure 1, both the germination potential and germination rate of six test plant species were affected under treatments with EMT and EMB. The germination potential of *Z. pacifica* was significantly reduced by EMT at concentrations ≥ 7.5 g/L, showing only 1.67% germination potential under 60 g/L of EMT. The germination potentials of *T. aestivum*, *L. sativa*, and *A. canina* were significantly inhibited by EMT at concentrations ≥ 30 g/L. When the applied concentration of EMT increased to 60 g/L, all the tested species showed significantly lower germination potential than controls (Figure 1A). The germination rates of *L. sativa* and *Z. pacifica* were significantly reduced under EMT concentrations ≥ 30 g/L, with IC_50_ values of 26.37 and 24.92 g/L, respectively (Table 1). After treatments with EMT of 60 g/L, significant reductions in germination rates were observed in case of all tested plants except for *T. aestivum* (Figure 1B).

Treatments of EMB at concentrations ≥ 7.5 g/L significantly inhibited the germination potential of all test species except for *A. canina*. Under EMB 60 g/L, the germination potentials of *L. sativa* and *Z. pacifica* were only 6.67% and 3.67%, and with corresponding IC_50_ values of 11.64 and 17.66 g/L, respectively (Figure 1C, Table 1). Significant decreases in germination rates of *L. sativa* and *Z. pacifica* were observed when treated with EMB at concentrations ≥ 15 g/L, and all target species displayed significantly lower germination rates than controls under 60 g/L EMT (Figure 1D). Both EMT and EMB exhibited consistent concentration-dependent inhibitory effects on seed germination of all test species, with stronger impacts on germination potential than germination rate.

### 2.2. Effects of EMT and EMB on Seedlings’ Growth

**Figure 2:**
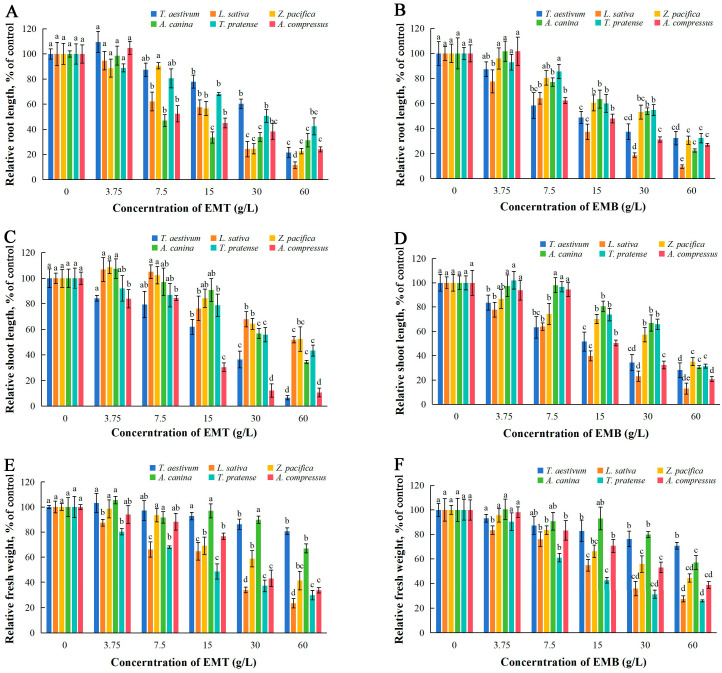
Seedling growth of six plants under treatments with EMT (**A**,**C**,**E**) and EMB (**B**,**D**,**F**). The results presented are the mean of three replicates ± SE of relative root length (**A**,**B**), relative shoot length (**C**,**D**), and relative fresh weight (**E**,**F**). Different letters denote significant differences at *p* < 0.05 according to one-way ANOVA with an LSD test.

EMT exhibited significant inhibitory effects on root length of *L. sativa*, *A. canina*, and *A. compressus* at concentrations ≥ 7.5 g/L, while all tested plant species showed markedly suppressed root growth under treatments with EMT at concentrations ≥ 15 g/L (Figure 2A). The IC_50_ values of EMT for root growth inhibition in *T. aestivum*, *L. sativa*, *Z. pacifica*, *A. canina*, *T. pratense*, and *A. compressus* were 32.02, 15.03, 19.57, 14.19, 36.71, and 9.96 g/L, respectively (Table 1). Similarly, after being treated with EMB at concentrations ≥ 15 g/L, the root growth of all test plant species was significantly inhibited (Figure 2B). The IC_50_ value of EMB for *L. sativa* root elongation was 10.44 g/L (Table 1).

The shoot growth of *A. compressus* was significantly reduced under treatments of all EMT concentrations. At high concentrations of 30 and 60 g/L, EMT significantly inhibited shoot growth in all species, with IC_50_ values of 17.42 and 12.08 g/L for *T. aestivum* and *A. compressus*, respectively (Figure 2C, Table 1). EMB treatments resulted in significant inhibition of shoot growth in *T. aestivum* and *L. sativa* at all concentrations, while EMB notably suppressed shoot growth in all six species at concentrations ≥ 15 g/L, with an IC_50_ of 11.21 g/L for *L. sativa* shoot elongation (Figure 2D, Table 1).

EMT significantly decreased fresh weight in *L. sativa* and *T. pratense* at all treatment levels, while the growth of all test species was markedly inhibited under the highest concentration of 60 g/L (Figure 2E). The IC_50_ values for fresh weight inhibition by EMT were 19.09 and 17.49 g/L for *L. sativa* and *T. pratense*, respectively (Table 1). EMB showed comparable phytotoxic effects on biomass accumulation with EMT, showing significant growth suppression at 30 and 60 g/L for all plants (Figure 2F). Notably, *L. sativa* fresh weight was reduced by EMB at all concentrations, with an IC_50_ of 19.67 g/L (Table 1).

### 2.3. LC-HRMS Level 3 Analysis of Active Compounds in Flowers of M. biondii

Based on LC-HRMS analysis, 120 and 99 secondary metabolites were annotated (putative assignment of metabolites based on spectral data (accurate mass or MS/MS matching to a database), without confirmation by an authentic reference standard or unambiguous structural elucidation) in EMT and EMB, respectively, including organic acids, phenolic acids, sugars, fatty acids, flavonoids, alkaloids, and others Table 2 and Table S1). There were 28 and 25 compounds with relative amounts ≥ 0.5% of all secondary metabolites in EMT and EMB, respectively. Among them, malic acid, D-(+)-mannose, sucrose, citric acid, 9-hydroxy-10,12-octadecadienoic acid, (15Z)-9,12,13-trihydroxy-15-octadecenoic acid, and chlorogenic acid were the compounds with relatively high abundance in both EMT and EMB (Table 2).

### 2.4. Joint Action of MA and CA

**Figure 3:**
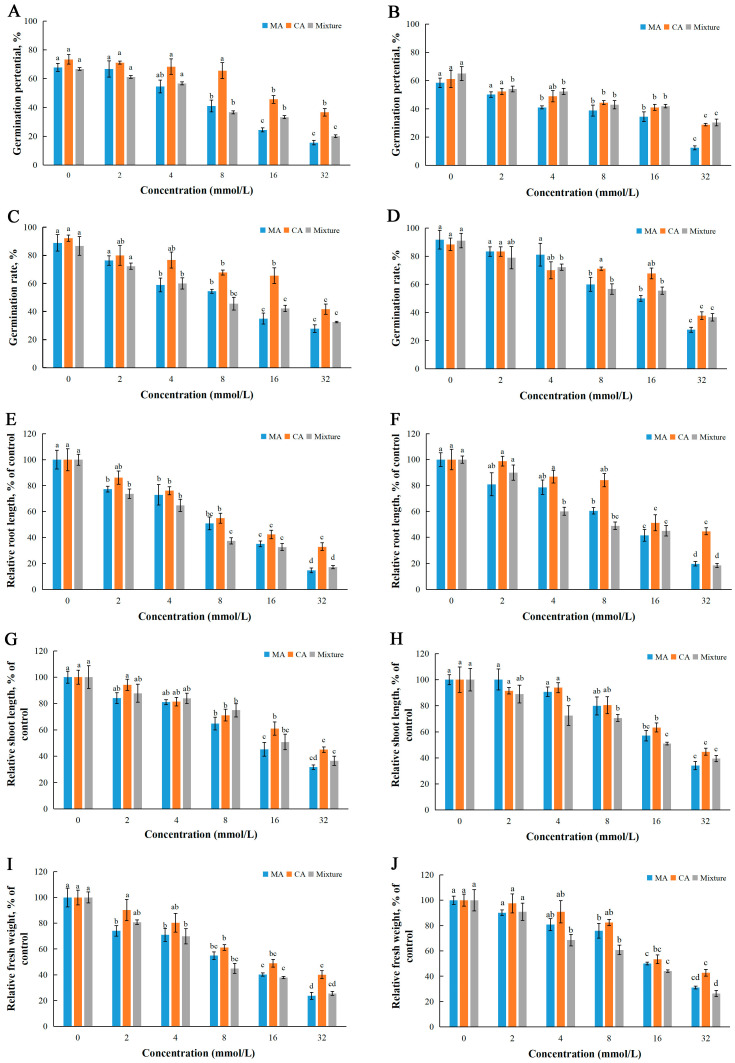
Seed germination and seedling growth of *L. sativa* (**A**,**C**,**E**,**G**,**I**) and *Z. pacifica* (**B**,**D**,**F**,**H**,**J**) under treatments with MA, CA, and mixture. The results presented are the mean of three replicates ± SE of germination potential (**A**,**B**), germination rate (**C**,**D**), relative root length (**E**,**F**), relative shoot length (**G**,**H**), and relative fresh weight (**I**,**J**). Different letters denote significant differences at *p* < 0.05 according to one-way ANOVA with an LSD test.

The relative concentration of MA and CA was high in both EMT and EMB, particularly, MA was the most abundant compound in EMB, with a proportion of 24.13% of all secondary metabolites (Table 2). The phytotoxic effects and joint action of MA and CA were evaluated using *L. sativa* and *Z. pacifica* as target plants. The results showed that MA and CA inhibited the seed germination and seedling growth of *L. sativa* and *Z. pacifica*, and the inhibition increased with the increase of treated concentrations. The mixture of MA and CA also displayed a concentration-dependent manner on the germination and growth of test seeds (Figure 3). MA showed stronger phytotoxic effects on the target seeds than CA. For seed germination, MA significantly reduced the germination rate and germination potential of the test seeds at concentrations ≥ 8 mmol/L, while the significant effects on seed germination of CA need higher concentrations (Figure 3A–D). For seedling growth, MA showed significant inhibitory effects on root length, shoot length, and fresh weight at concentrations more than 4 or 8 mmol/L in most cases, while the concentrations of CA that had significant phytotoxic effects on plant growth were more than 8 or 16 mmol/L (Figure 3E–J). Particularly, MA at the lowest test concentration of 2 mmol/L has already exerted a significant reduction on root elongation and biomass of *L. sativa* (Figure 3G,I). The inhibition of the mixture was often ranging between MA and CA, and sometimes was the strongest. For instance, the IC_50_ values of MA, CA, and the mixture for *L. sativa* germination potential were 11.81, 29.21, and 14.15 mmol/L, respectively, while for *Z. pacifica* root length was 10.76, 22.54, and 9.03 mmol/L, respectively. According to ADM analysis, the mixture of MA and CA showed all synergistic joint action on seed germination and seedlings growth indicators of *L. sativa* and *Z. pacifica*, except the additive activity of *Z. pacifica* germination potential (Table 3).

## 3. Discussion

Plants of the genus *Magnolia*, as a relatively primitive group within angiosperms, not only possess exceptional ornamental value but also exhibit noteworthy allelopathic properties in ecosystems and have attracted significant scientific interest [36]. After blooming in spring, the barks and petals of the flowers of *M. biondii* successively fall. Then the secondary metabolites in the flowers were released into the surrounding environment. Among them, the allelochemicals could affect the growth and development of neighboring plants. In this study, several crops, lawn, and garden plants, which likely coexist in the same habitat with *M. biondii*, were selected as the receptor plants. Like many *Magnolia* species, the flowering period of *M. biondii* occurs in early spring, about mid-March in Central China, when surrounding plants are germinating or in the seedling stage [1,4]. Both EMT and EMB exhibited significant inhibitory effects on seed germination and seedling growth of receptor plants. As an important mode of plant interaction, allelopathy plays a critical role in shaping community structure, species distribution, and succession processes within ecosystems [37]. Therefore, the results suggested that the flower litter from *M. biondii* may substantially influence the growth of surrounding plants and have the potential to affect community structure and function during ecosystem development processes.

Plant litter, comprising dead branches, fallen leaves, flowers, and senescent root systems generated during plant growth and development, serves as a vital component in ecosystem material cycling and energy flow [28]. Through its inherent chemical composition and properties, plant litter can stably and continuously supply nutrients essential for plant growth, effectively maintain soil moisture, significantly reduce temperature fluctuations in the topsoil layer, profoundly improve soil texture and structure, and comprehensively create favorable habitats for plant regeneration [38]. Concurrently, during decomposition, various allelochemicals are released from litter, which regulate the growth, development, and distribution of surrounding organisms. Allelochemicals can influence physiological and biochemical processes, cell division, and gene expression of receptor plants [25]. Aqueous methanol extracts of red pine litter inhibited the growth of cress (*Lepidium sativum*) and *Digitaria sanguinalis* L., and increasing the extract concentration increased the inhibition. Two main inhibitory substances, 9*α*,13*β*-epidioxyabeit-8(14)en-18-oic acid and abscisic acid-*β*-D-glucopyranosyl ester, were isolated from the litter [39]. Leaf litter of *Leucaena leucocephala* induced inhibitory effects on germination and growth of two forest crops and three agricultural crops, and the effects depended on the concentration of litter extract and type of receptor species [40]. Leaf litter of *Cinnamomum septentrionale* Hand.-Mazz significantly inhibited the growth and photosynthesis of *Eucalyptus grandis* Hill ex Maid saplings, and the inhibition strengthened with increasing concentrations [41]. High concentration of *Juniperus rigida* litter aqueous extract (0.10 g DW/mL) significantly inhibited the seed germination and seedling growth of wheat and *Pinus tabuliformis* [42]. Together with this study, these results demonstrated that the allelopathic effects of plant litter widely exist in both natural and artificial ecosystems, influencing ecological processes at multiple levels. The allelochemicals in litter significantly affect community structure, interspecific relationships, and soil ecosystems, thereby playing crucial roles in agricultural and forestry practices.

Many secondary metabolites were assigned from the flower litter of *M. biondii* in this study. Among them, there were several known allelochemicals that displayed strong phytotoxic effects on target plant species. The study of the effects of chlorogenic acid on germination and the physiological response in *Festuca arundinacea* showed that all treatments conferred significant reduction in germination index, seed vigor, fresh seedling weight, coleoptile length, primary root length, and root hairs compared to the control [43]. Reduction in germination rate, root, and stem growth was observed in wild poinsettia (*Euphorbia heterophylla*) and morning glory (*Ipomoea grandifolia*) after treatments with pure aconitic acid [44]. *Trans*-aconitic acid at the concentrations of 2.5–10 mmol/L affected the root system and photosynthetic apparatus of *Glycine max*, resulting in significant inhibition of the plant growth [45]. Azelaic acid inhibited root growth, stimulated lateral and adventitious roots, and altered the root apical meristem by reducing meristem cell number, length, and width of *Arabidopsis thaliana*. The treatments also slowed down the roots’ gravitropic response, likely due to a reduction in statoliths, starch-rich organelles involved in gravity perception, as well as a reduction in auxin transport and its distribution into the meristematic zone [46]. The allelopathic effects of EMT and EMB on the six test plants were dependent on their allelochemicals. Both EMT and EMB displayed inhibitory effects on seed germination and seedling growth of target plants. However, there were some differences in the strengths of EMT and EMB on certain plants or growth indicators. For example, EMT displayed stronger inhibitory effects on the shoot growth of *T. aestivum* than EMB at the highest concentration of 60 g/L, while the germination potential of *L. sativa* was more sensitive to EMB than EMT under treatments with 7.5 and 15 g/L. These results were likely due to the differences in the types of allelochemicals in EMT and EMB, as well as variations in the concentrations of annotated allelochemicals between the two materials. Additionally, different plant species and various growth indicators exhibit different sensitivities to the same allelochemical. Besides the known allelochemicals, there may be other phytotoxic compounds in the flower litter of *M. biondii*, and additional studies are needed to conduct comparative analyses between the aqueous extracts and those obtained using various organic solvents (e.g., ethanol, petroleum ether, dichloromethane, ethyl acetate) to characterize their phytochemical profiles.

In natural ecosystems, various allelochemicals often coexist, and the allelopathic activities are largely attributed to the presence of several compounds in a mixture. Therefore, the joint action of the allelochemicals is key to understanding the ecological impact of allelopathy. Previous studies showed that different kinds and proportions of allelochemicals displayed different joint actions. The joint action of the phenolic acids, p-hydroxybenzoic, p-coumaric, and ferulic acids, on the root growth inhibition of perennial ryegrass (*Lolium perenne* L.) was evaluated by the additive dose model (ADM), and the results showed that there were no synergistic activities of phenolic acids in the mixture [47]. The joint action on *L. perenne* and *Myosotis arvensis* root growth of binary and ternary mixtures of the main allelochemicals in wheat, benzoxazinone derivatives, and phenolic acids was studied using ADM. Results showed that binary mixtures of phenolic acids responded predominantly additively on both plant species, while binary and ternary mixtures of benzoxazinone derivatives and phenolic acids responded primarily antagonistically [48]. The allelopathic interactions among five representative allelochemicals (coumarin, *ρ*-hydroxybenzoic acid, protocatechuic acid, stearic acid, and *ρ*-aminobenzene-sulfonic acid) at different proportions were studied using *Microcystis aeruginosa* as the test receptor. Results showed that binary or ternary mixtures of allelochemicals obtained three types of allelopathic interactions, including synergistic, antagonistic, and additive effects, while combinations of four or five allelochemicals only yielded antagonistic effects [49].

MA and CA are the main secondary metabolites in EMT and EMB with high relative concentrations. Consistent with this study, previous studies showed that MA and CA displayed phytotoxic effects on test plants. Through a bioassay-guided isolation process from the leaf extracts of *Tamarindus indica* L., MA and CA were identified as potential allelochemicals, which demonstrated allelopathic effects on the growth of lettuce seedlings [50]. Melon seedlings treated with MA showed significantly lower fresh weight compared with the control, while CA inhibited radicle growth at low concentrations and inhibited hypocotyl growth at high concentrations [51]. MA and CA inhibited seed germination rate and the growth of radicle and shoot of *Brassica chinensis* L., resulting in damage to the seed cell membranes [52]. The joint action of MA and CA on seed germination and seedling growth of *L. sativa* and *Z. pacifica* had a predominantly synergistic effect. The joint effects of different allelochemicals depend on various factors such as the allelochemicals used, their respective proportions, the concentrations of the mixture, as well as the receptor species. The joint action of a binary combination of MA and CA in EMT and EMB likely played an important role in the allelopathy of the flower litter of *M. biondii.*

## 4. Materials and Methods

### 4.1. Materials

The freshly fallen flowers’ litter (tepals and bracts) were immediately collected from several *M. biondii* trees on the campus of Nanyang Normal University on 12 March 2024. About 1000 g of flower litter was dried in the dark at room temperature and then stored at 4 °C before use. Seeds of *Triticum aestivum* L. and *Lactuca sativa* L. were purchased from Henan Fengtian Seed Industry Co., Ltd. (Zhengzhou, China). Seeds of *Zoysia pacifica*, *Agrostis canina* L., *Trifolium pratense* L., and *Axonopus compressus* were purchased from Nanyang Wosen Landscaping Industry Co., Ltd. (Nanyang, China). Malic acid (MA) and citric acid (CA) with HPLC levels were purchased from Alfa Aesar Inc. (Shanghai, China).

### 4.2. Aqueous Extracts of M. biondii Flower Litter

The dried tepals and bracts of *M. biondii* were crushed into fine powder. Then, 1 L of distilled H_2_O was added to 60 g of the powder and extracted with shaking at 25 °C for 48 h. The extracts were filtered with filter paper and sterilized by a 0.22 μm water-based filter membrane to obtain an aqueous extract with a concentration of 60 g/L, which was diluted with sterile water to 30, 15, 7.5, and 3.75 g/L, respectively. Aqueous extracts of *M. biondii* tepal (EMT) and bract (EMB) were stored at 4 °C before use.

### 4.3. Seed Germination

Uniformly sized seeds were surface-sterilized using 2% NaClO, followed by 5 rinses with sterile distilled H_2_O. The sterilized seeds were transferred to a Petri dish (*Φ* = 9 cm) containing filter paper with 50 seeds in each dish. EMT or EMB (3 mL) with different concentrations was added to the Petri dish, and sterile distilled H_2_O was used as a control. Seeds were cultured in darkness at 25 ± 1 °C with a humidity of 60 ± 5%, and the number of germinated seeds was recorded every 24 h. The germination potential and germination rate of seeds were calculated according to the following formulas.

Germination potential = (number of germinated seeds in the germination peak period/total number of the subjects’ seeds) × 100%

Germination rate = (final number of germinated seeds/total number of the subjects’ seeds) × 100%

### 4.4. Seedling Growth

The sterilized seeds were transferred to filter paper soaked with 3 mL sterile distilled H_2_O in a Petri dish (*Φ* = 9 cm) and germinated at 25 ± 1 °C in darkness with a humidity of 60 ± 5%. After germination, at least 6 seedlings of similar size were transferred to every well of a 6-well plate (Thermo Fisher Scientific (Suzhou) Instruments Co., Ltd., Suzhou, China), which contained various concentrations of EMT or EMB and sterile distilled H_2_O as a control. After incubating under the same condition of germination conditions for 48 h, the root and shoot length of the seedlings were measured using a ruler, and the fresh weight was measured by an analytical balance. The relative growth of the seedlings was calculated as the ratio of the treated groups to the control.

### 4.5. LC-HRMS

HPLC analysis was performed on UltiMate 3000 C18 column (100 mm × 2.1 mm, 1.9 μm, Thermo Fisher Scientific, Waltham, MA, USA). The mobile phase consisted of two solvents: solvent (A) deionized water with 1% formic acid, and solvent (B) acetonitrile with 1% formic acid.

Gradient elution was performed at a flow rate of 0.3 mL/min at room temperature. Elution profile was isocratic from 0 to 10 min (90% (A), 10% (B)), from 10 to 17 min (0% (A), 100% (B)), from 17 to 20 min, isocratic (90% (A), 10% (B)).

The high-resolution mass spectrometry analysis was performed using Q-Exactive Orbitrap (Thermo Fisher Scientific, Waltham, MA, USA) equipped with a heated electrospray ionization (HESI) source. The ion source temperature was set to 310 °C, and the capillary temperature was maintained at 320 °C. The sheath gas and auxiliary gas flow rates were 30 and 10 L/min, respectively. The spray voltages were configured as 3.0 kV for positive ion mode and 2.8 kV for negative ion mode. Data-dependent acquisition (DDA) was employed with a loop count of 10. Higher-energy collisional dissociation (HCD) was performed using step-wise normalized collision energies (NCE) of 10, 28, and 35 eV. Full MS scans (MS^1^) were acquired over a range of *m*/*z* 80–1200 with a resolution of 70,000, an automatic gain control (AGC) target of 3 × 10^6^, and an injection time of 200 ms. For tandem MS (MS^2^), the resolution was set to 17,500, with an AGC target of 1 × 10^5^ and an injection time of 50 ms. Data were processed by Compound Discover 3.2 (Thermo Fisher Scientific, CA, USA) and compared with databases including ChEMBL (https://www.ebi.ac.uk/chembl/, accessed on 23 September 2024), ChEBI (https://www.ebi.ac.uk/chebi/init.do, accessed on 23 September 2024), PubChem (https://pubchem.ncbi.nlm.nih.gov/, accessed on 23 September 2024), and mzCloud (https://www.mzcloud.org/, accessed on 23 September 2024) to annotate compounds. All compounds have been tentatively assigned based on level 3 annotation, which refers to tentative assignment of metabolites based solely on spectral data, without confirmation by an authentic reference standard or unambiguous structural elucidation [53].

### 4.6. Joint Action of Allelochemicals

MA and CA were dissolved in distilled H_2_O with concentrations of 2, 4, 6, 8, and 10 mmol/L, respectively. The concentrations of the mixture of MA and CA with a ratio of 1:1 were as same as the single compound. Bioassays of seed germination and seedling growth of *L. sativa* and *Z. pacifica* were carried out as described above. The joint action of MA and CA was evaluated using the additive dose model (ADM) [47]. The relative potency (*r*) between the allelochemicals was calculated as follows:

*r* = IC_50MA_/IC_50CA_

The predicted IC_50_ of the mixtures was calculated as follows:

EC_50MAmix_ = IC_50MA_/(*α* + (1 − *α*)*r*)

where *α* is the ratio of MA in the mixture.

The ADM isoboles were constructed according to Inderjit et al. [47]. Significant deviations from ADM were termed as antagonism (higher) or synergism (lower); others were termed as addition.

### 4.7. Statistical Analysis

The bioassays were conducted with three replicates for each treatment. The data were evaluated by one-way ANOVA with the least significant differences (LSD) test at the 0.05 significance level using SPSS 22.0 (SPSS Inc., Chicago, IL, USA). The concentrations required for 50% inhibition in the assay (defined as IC_50_) of the extracts and allelochemicals on seed germination and seedling growth of target plants were determined using SPSS 22.0 software.

## 5. Conclusions

In this study, we first reported the allelopathy and the potential allelochemicals of the *M. biondii* flower litter. We found that EMT and EMB exhibited inhibitory effects on the seed germination and seedling growth of the six test plants. The inhibition of germination potential, germination rate, root and stem growth, and biomass increased with the increase of the treated concentrations of EMT and EMB, showing a concentration-dependent manner. The known allelochemicals, such as organic acids, phenolic acids, and flavonoids with strong phytotoxic effects, likely contribute to the allelopathy of *M. biondii* flower litter. Among them, MA and CA were with high relative concentrations, and their joint action predominantly showed synergistic effects, which may play a potential role in the allelopathic interaction between *M. biondii* and surrounding plants. These findings provide a valuable scientific basis for evaluating the potential of the *Magnolia* genus and suggest that the flower litter of *M. biondii* could be considered as an efficient source of allelochemicals. Nevertheless, further studies are needed to identify other potential allelochemicals in EMT, EMB, and organic solvent extracts of *M. biondii* flowers, and to investigate their ecological roles under natural conditions.

## Figures and Tables

**Table 1:** IC_50_ values (g/L) of EMT and EMB on seed germination and seedling growth of target plants.

Materials	Test Plants	Germination Potential	Germination Rate	Root Length	Stem Length	Fresh Weight
EMT	*T. aestivum*	41.69	–	32.02	17.42	–
	*L. sativa*	25.22	26.37	15.03	68.89	19.09
	*Z. pacifica*	17.75	24.92	19.57	60.71	39.94
	*A. canina*	37.62	66.33	14.19	39.99	–
	*T. pratense*	46.79	–	36.71	44.03	17.49
	*A. compressus*	67.85	–	9.96	12.08	31.31
EMB	*T. aestivum*	–	–	17.61	16.85	–
	*L. sativa*	11.64	27.82	10.44	11.21	19.67
	*Z. pacifica*	17.66	21.62	28.65	34.77	39.8
	*A. canina*	32.28	72.66	25.4	39.76	–
	*T. pratense*	42.01	–	30.46	38.3	15.11
	*A. compressus*	36.97	–	13.52	20.72	35.48

–, the IC_50_ was more than 100 g/L and unable to determine accurate values based on the existing data.

**Table 2:** LC-HRMS annotation of main secondary metabolites from EMT and EMB.

No.	Proposed Compounds	Molecular Formula	MW	Mass Error (ppm)	Main Fragment MS2	RT (min)	Peak Area (%)	
							EMT	EMB
1	Asparagine	C_4_H_8_N_2_O_3_	132.0532	−2	88.0403, 72.009	0.85	0.42	0.55
2	Choline	C_5_H_13_NO	103.0993	−3.72	60.0809, 58.0650	0.861	3.16	0.14
3	Mannitol	C_6_H_14_O_6_	182.0788	−1.5	181.0722, 180.0597	0.868	0.29	0.77
4	Raffinose	C_18_H_32_O_16_	504.1678	−0.39	504.165, 503.1616, 543.1309	0.871	0.02	0.52
5	Sucrose	C_12_H_22_O_11_	342.1158	−1.23	179.056, 119.0348	0.878	10.32	16.03
6	Adonitol	C_5_H_12_O_5_	152.0682	−2.13	89.0243, 101.0243, 59.0138	0.895	0.48	0.92
7	Trigonelline HCl	C_7_H_7_NO_2_	137.0473	−3.06	137.0445, 136.0616	0.901	2.00	0.11
8	4-Guanidinobutyric acid	C_5_H_11_N_3_O_2_	145.0847	−3.02	88.0759, 104.1834, 146.1761	0.924	0.59	0.01
9	Quebrachitol	C_7_H_14_O_6_	194.0787	−1.87	217.0799, 411.1486	0.94	0.41	1.19
10	Shikimic acid	C_7_H_10_O_5_	174.0525	−1.85	173.0049, 175.0611	0.946	0.57	2.64
11	Malic acid	C_4_H_6_O_5_	134.0213	−1.51	115.0036, 71.0137	0.96	10.65	24.13
12	D-(+)-Mannose	C_6_H_12_O_6_	180.0632	−1.14	127.0626	0.982	15.87	22.36
13	Citric acid	C_6_H_8_O_7_	192.0268	−0.86	111.0086, 87.00864	1.002	3.91	6.03
14	Arabinofuranosyluracil	C_9_H_12_N_2_O_6_	244.0691	−1.65	245.0431, 110.0246, 189.2093	1.002	0.47	0.60
15	4-Oxoproline	C_5_H_7_NO_3_	129.0425	−1.12	55.0188, 82.0298, 99.9256	1.015	0.40	0.57
16	Aconitic acid	C_6_H_6_O_6_	174.0162	−1.35	129.0192, 111.0087, 85.0294	1.107	0.83	0.20
17	L-Leucine	C_6_H_13_NO_2_	131.0942	−2.96	132.1018, 86.0963, 69.0699, 70.0651	1.202	0.67	0.01
18	D-(+)-Glucose	C_6_H_12_O_6_	180.0629	−2.22	179.056, 101.0234, 71.0137, 161.0454, 143.0343	1.343	0.79	2.71
19	Chlorogenic acid	C_16_H_18_O_9_	354.0949	−0.61	193.0494, 164.0418, 107.049	3.087	6.13	1.77
20	Scopolin	C_16_H_18_O_9_	354.0949	−0.28	192.0595, 179.0347, 165.0511, 150.0345, 149.0583, 137.0573	3.179	0.21	0.79
21	Caffeic acid	C_9_H_8_O_4_	180.0422	−0.3	179.0349, 134.037, 135.0450, 92.9199	4.304	0.50	0.14
22	Echinacoside	C_35_H_46_O_20_	786.2571	−1.45	161.0243, 477.1613, 162.0277	4.571	0.18	0.80
23	Magnoflorine	C_20_H_23_NO_4_	341.1617	−3.03	297.113, 265.0851,	4.778	1.71	1.06
24	p-Hydroxybenzaldehyde	C_7_H_6_O_2_	122.0367	−0.89	121.0294, 92.2206,	4.857	0.73	1.10
25	Purpureaside C	C_35_H_46_O_20_	786.2571	−1.47	471.1538, 785,623	5.066	1.23	0.25
26	2-Hydroxycinnamic acid	C_9_H_8_O_3_	164.0472	−0.7	116.9285, 120.0534, 121.0292	5.559	0.88	0.15
27	Rutin	C_27_H_30_O_16_	610.1530	−0.68	300.0274, 271.0249	5.588	2.08	0.59
28	Lariciresinol 4-*O*-glucoside	C_26_H_34_O_11_	522.2106	−1.03	329.1392, 359.1498	5.781	0.79	0.28
29	Isoacteoside	C_29_H_36_O_15_	624.2048	−1.02	623.1974, 461.1665	5.84	2.14	0.38
30	Poliumoside	C_35_H_46_O_19_	770.2622	−1.42	162.0277, 161.0234	5.911	3.49	0.86
31	Pinoresinol 4-*O*-glucoside	C_26_H_32_O_11_	520.1941	−0.71	357.1343,151.0399	6.183	0.70	0.35
32	Azelaic acid	C_9_H_16_O_4_	188.1047	−0.63	187.0974, 99.9256	6.74	2.69	0.71
33	Tiliroside	C_30_H_26_O_13_	594.1370	−0.6	284.0324, 255.0301, 227.0346, 145.0293	7.431	0.01	2.28
34	Abscisic acid	C_15_H_20_O_4_	264.1361	−0.1	219.1389, 204.1154	7.53	0.73	0.07
35	(15*Z*)-9,12,13-Trihydroxy-15-octadecenoic acid	C_18_H_34_O_5_	330.2406	−0.2	171.1026, 229.1442, 127.1127, 211.134	8.764	5.38	1.06
36	(10*E*,12*Z*)-9-Hydroxyoctadeca-10,12-dienoic acid	C_18_H_32_O_4_	312.2300	−0.33	310.1715, 293.2123,	11.634	1.42	0.16
37	Veraguensin	C_22_H_28_O_5_	372.1915	−5.91	151.0750, 179.1062, 217.1217	12.053	1.07	0.01
38	9-Hydroxy-10,12-octadecadienoic acid	C_18_H_32_O_3_	296.2348	−1.16	295.2275, 277.2171, 195.1387	12.895	7.03	2.65
39	13S-Hydroxy-6*Z*,9*Z*,11*E*-octadecatrienoic acid	C_18_H_30_O_3_	294.2194	−0.27	147.0972, 125.097	13.547	0.27	0.59

**Table 3:** IC_50_ values (mmol/L) and joint action of MA and CA on seed germination and seedling growth of *L. sativa* and *Z. pacifica*.

Test Plants	Growth Indicators	IC_50_ Values of MA	IC_50_ Values of CA	IC_50_ Values of Mixture	Joint Action of MA and CA
*L. sativa*	Germination potential	11.81	29.21	14.15	synergism
	Germination rate	11.54	33.56	13.54	synergism
	Root length	8.06	12.43	6.23	synergism
	Stem length	14.34	24.59	18.78	synergism
	Fresh weight	9.35	17.01	8.8	synergism
*Z. pacifica*	Germination potential	13.86	34.32	30.69	addition
	Germination rate	16.6	32.57	20.04	synergism
	Root length	10.76	22.54	9.03	synergism
	Stem length	20.05	27.19	18.41	synergism
	Fresh weight	16.78	22.32	11.86	synergism

## Data Availability

Data are contained within the article and Supplementary Materials.

## Supplementary Materials

The following supporting information can be downloaded at: https://www.mdpi.com/article/10.3390/plants14111577/s1, Table S1: LC-HRMS annotation of secondary metabolites with relative concentrations < 0.5% in both EMT and EMB.

## Abbreviations

The following abbreviations are used in this manuscript:

EMT	aqueous extracts of *M. biondii* flower tepal litter
EMB	aqueous extracts of *M. biondii* flower bract litter
LC-HRMS	liquid chromatography high-resolution mass spectrometry
MA	malic acid
CA	citric acid
ADM	additive dose model
