# Supplementary material for: Phytotoxic Effects of the Aqueous Extracts of Magnolia biondii Pamp. Flower Litter and the Joint Action of Allelochemicals
Source: Plants (Basel). 2025 May 22;14(11):1577. doi: 10.3390/plants14111577 (PMC12158015; doi:10.3390/plants14111577)
Supplement: Supplementary file 1 [file plants-14-01577-s001.zip › plants-3594109-supplementary.pdf]

Table S1. LC-HRMS annotation of secondary metabolites with relative concentrations < 0.5% in both EMT and EMB.

No.	Proposed Compounds	Molecular Formula	MW	Mass Error (ppm)	Main Fragment MS2	RT (min)	Peak Area (%)	
							EMT	EMB
1	L-histidine	$C_6H_9N_3O_2$	155.069	-2.57	136.076, 93.0446	0.805	0.02	<0.01
2	DL-arginine	$C_6H_{14}N_4O_2$	174.111	-2.77	158.092, 175.119, 130.097, 116.07	0.806	0.35	<0.01
3	D-glucosamine	$C_6H_{13}NO_5$	179.079	-2.92	162.076, 162.055, 84.0443	0.816	0.04	<0.01
4	nystose	$C_{24}H_{42}O_{21}$	666.221	-0.6	179.056, 383.119	0.845	0.11	0.22
5	D-glutamine	$C_5H_{10}N_2O_3$	146.069	-2.32	104.07, 102.0548, 128.081	0.853	0.05	0.06
6	gamma-aminobutyric acid	$C_4H_9NO_2$	103.063	0.27	104.1068, 87.0439, 69.0335, 86.06	0.872	0.13	<0.01
7	cytidine	$C_9H_{13}N_3O_5$	243.085	-3.49	133.049, 69.0447	0.883	0.07	<0.01
8	cytosine	$C_4H_5N_3O$	111.043	-3.24	95.0238, 69.0446, 94.0399, 71.1535	0.888	0.09	<0.01
9	adenine	$C_5H_5N_5$	135.054	-2.52	119.035, 92.0245	0.897	0.47	0.02
10	2-pyrrolidinecarboxylic acid	$C_5H_9NO_2$	115.063	-3.01	70.0651, 116.7633	0.905	0.42	0.01
11	(2R)-2,3-dihydroxypropanoic acid	$C_3H_6O_4$	106.026	-2.41	75.0086, 59.0138	0.921	0.22	0.25
12	isomaltulose	$C_{12}H_{22}O_{11}$	342.116	-0.58	179.056,89.0234, 71.0137, 59.0138	0.956	0.08	/
13	isoguanosine	$C_{10}H_{13}N_5O_5$	283.091	-1.28	152.0564, 135.03, 283.14, 112.087	0.979	0.10	0.14
14	niacinamide	$C_6H_6N_2O$	122.048	-3.37	122.096, 121.065,	0.987	0.03	<0.01
15	guanosine	$C_{10}H_{13}N_5O_5$	283.091	-3.04	282.0852, 151.075	1.155	0.06	/
16	L-tyrosine	$C_9H_{11}NO_3$	181.074	-2.15	136.086, 165.0542, 119.0484	1.155	0.04	/
17	guanine	$C_5H_5N_5O$	151.049	-2.32	110.35, 135.03	1.156	0.05	/
18	N-acetyltyramine	$C_{10}H_{13}NO_2$	179.094	-2.6	130.67, 116.20, 131.22, 156.90, 173.19	1.159	0.02	/
19	L-phenylalanine	$C_9H_{11}NO_2$	165.079	-2.39	120.082, 103.0554, 149.0621, 133.0622, 130.098	1.495	0.29	/
20	forsythoside E	$C_{20}H_{30}O_{12}$	462.173	-0.77	321.1004, 237.0045	1.68	0.09	0.28
21	valeramide	$C_5H_{11}NO$	101.084	-2.43	72.0807, 58.0653	1.735	/	0.16
22	vanillic acid	$C_8H_8O_4$	168.042	-2.12	151.038, 123.41, 65.0386	1.959	0.04	/
23	protocatechuic acid	$C_7H_6O_4$	154.027	-0.61	109.0294, 108.395, 108.088	2.043	0.10	0.17
24	esculin	$C_{15}H_{16}O_9$	340.079	-1.38	335.72, 202.38, 223.28	2.197	0.01	0.15
25	tryptophan	$C_{11}H_{12}N_2O_2$	204.09	-0.3	42.066, 116.05, 74.0246	2.464	0.18	0.11
26	L-tryptophan	$C_{11}H_{12}N_2O_2$	204.089	-2.13	159.093, 142.066, 204.086, 116.928	2.472	0.42	0.01

27	BMK methyl glycidate	$C_{11}H_{12}O_3$	192.078	-2.06	118.041, 79.0542, 161.0592, 143.0604, 55.018	2.861	0.14	0.01
28	eleutheroside B/Syringin	$C_{17}H_{24}O_9$	372.141	-2.68	161.0594, 163.0758	2.873	0.08	<0.01
29	4-pentylaniline	$C_{11}H_{17}N$	163.136	-1.62	117.0332, 145.0282, 89.0385, 107.0491, 79.0542, 136.0474	2.984	0.04	0.01
30	3-hydroxy-1-(4-hydroxyphenyl)propan-1-one	$C_9H_{10}O_3$	166.063	-0.87	165.056, 164.895	3.292	0.04	0.03
31	2-isopropyl malic acid	$C_7H_{12}O_5$	176.068	-0.69	115.0398, 175.061	3.309	0.16	0.13
32	salicylic acid	$C_7H_6O_3$	138.032	-1.13	93.0344, 137.0243, 108.0214	3.453	0.14	0.17
33	skimmin	$C_{15}H_{16}O_8$	324.083	-3.76	145.028, 117.033, 135.044	4.929	0.01	<0.01
34	plantamajoside	$C_{29}H_{36}O_{16}$	640.2	-1.14	477.162, 162.028, 398.80, 113.02	4.974	0.09	0.37
35	3-*O*-feruloyl quinic acid	$C_{17}H_{20}O_9$	368.11	-0.83	191.056, 134.037, 173.045, 93.0344	5.094	0.11	0.04
36	isoforsythiaside	$C_{29}H_{36}O_{15}$	624.205	-0.98	623.197, 624.201, 345.314	5.177	0.15	0.42
37	2-adamantanone	$C_{10}H_{14}O$	150.104	-2.55	69.0334, 81.0699, 109.0644	5.259	0.02	/
38	N-acetylphenylalanine	$C_{11}H_{13}NO_3$	207.089	-0.95	164.0717, 58.0297, 91.0552	5.618	0.12	0.09
39	3-phenyllactic acid	$C_9H_{10}O_3$	166.063	-0.87	166.059, 72.993,116.929	5.754	0.08	0.05
40	isoscopoletin	$C_{10}H_8O_4$	192.042	-0.31	193.049, 178.026, 133.065	5.796	0.04	0.19
41	hyperoside	$C_{21}H_{20}O_{12}$	464.095	-0.4	301.034, 151.004	5.809	0.08	0.03
42	calceolarioside A	$C_{23}H_{26}O_{11}$	478.147	-0.58	477.14, 230.8540	5.818	0.06	0.18
43	L-valine	$C_5H_{11}NO_2$	117.079	-3.3	118.086, 59.0371, 72.0806	5.914	0.03	/
44	isoferulic acid	$C_{10}H_{10}O_4$	194.058	-1.35	178.0271,134.037,135,04	5.922	0.06	0.02
45	nicotiflorin	$C_{27}H_{30}O_{15}$	594.158	-0.73	285.034, 593.151, 255.03	5.98	0.13	0.05
46	narcissoside	$C_{28}H_{32}O_{16}$	624.169	-0.81	315.051, 315.045	6.032	0.02	<0.01
47	N-acetyltryptophan	$C_{13}H_{14}N_2O_3$	246.1	-0.48	74.0246, 116.035, 99.9257, 180.9894	6.091	0.01	0.09
48	astragalin	$C_{21}H_{20}O_{11}$	448.1	-0.6	461.1667, 284.1, 255.2	6.216	0.01	0.14
49	9-(2,3-dihydroxypropoxy)-9-oxononanoic acid	$C_{12}H_{22}O_6$	262.142	-0.44	125.097, 126.1, 169.087	6.322	0.17	0.03
50	cinnamic acid	$C_9H_8O_2$	148.052	-0.32	147.045, 102.949	6.382	0.14	/
51	2-hydroxy-3,	$C_9H_{10}O_5$	198.053	-0.07	182.025, 123.009,	6.523	0.04	/

	4-dimethoxy benzoic Acid				166.998,			
52	4-indolecarb aldehyde	C_9H_7NO	145.052	-2.93	118.0645, 98.0963, 88.0756, 144.101	6.598	0.02	<0.01
53	perillene	$C_{10}H_{14}O$	150.104	-2.55	150.1274, 134.096, 105.069, 93.0697, 81.0699, 69.0334	7.028	0.02	<0.01
54	4-hydroxybe nzoic acid	$C_7H_6O_3$	138.032	-1.13	138.02, 138.028, 108.021	7.104	0.06	0.09
55	pinoresinol dimethyl ether	$C_{22}H_{26}O_6$	386.171	-4.13	351.159, 298.119, 201.091	7.638	0.07	/
56	pinoresinol	$C_{20}H_{22}O_6$	358.142	-0.14	323.1992, 227.0683, 144.0148	7.868	0.17	0.01
57	syringaresin ol	$C_{22}H_{26}O_8$	418.162	-0.67	417.1554, 387.106, 181.051	8.097	0.03	<0.01
58	corchorifatty acid F	$C_{18}H_{32}O_5$	328.225	-0.27	328.221, 327.2715	8.345	0.33	0.20
59	luteolin	$C_{15}H_{10}O_6$	286.048	-0.2	285.0412, 151.004	8.496	<0.01	0.31
60	(12*Z*)-9,10,11 -trihydroxyo ctadec-12-en oic acid	$C_{18}H_{34}O_5$	330.24	-1.82	173.117, 67.0542, 81.0698, 109.101, 93.0698	8.763	0.05	/
61	phillygenin	$C_{21}H_{24}O_6$	372.156	-4.71	337.143, 284.104	9.16	0.08	<0.01
62	(-)-caryophyl lene oxide	$C_{15}H_{24}O$	220.182	-3.93	221.19, 119.085, 105.07	9.318	0.26	/
63	epimagnolin B	$C_{23}H_{28}O_7$	416.181	-5.04	151.0750, 328.13, 313.106	9.994	0.32	0.03
64	epiyangambi n	$C_{24}H_{30}O_8$	446.192	-4.88	181.0860, 429.1912, 411.1826,380.1642, 358.1454	10.111	0.19	0.01
65	dehydrodiis oeugenol	$C_{20}H_{22}O_4$	326.15	-5.7	203.106, 188.83, 137.059, 171.08	10.334	0.03	/
66	6-gingerol	$C_{17}H_{26}O_4$	294.183	0.34	294.179, 89.0245	10.507	0.03	0.03
67	eucalyptol	$C_{10}H_{18}O$	154.135	-3.24	137.132, 67.0543	10.583	0.12	/
68	aschantin	$C_{22}H_{24}O_7$	400.15	-5.41	173.059, 185.059, 136.086, 231.101, 219.101, 312.098	10.706	0.04	/
69	eugenol	$C_{10}H_{12}O_2$	164.083	-4.85	137.0594, 105.07, 119.0527, 147.0801	10.853	0.06	<0.01
70	ferulaldehyd e	$C_{10}H_{10}O_3$	178.062	-4.91	147.08, 148.088, 164.083	11.173	0.04	<0.01
71	*β*-asarone	$C_{12}H_{16}O_3$	208.109	-5.29	194.093, 181.085, 168.078, 179.07, 151.075	11.52	0.46	0.01
72	butyl benzoate	$C_{11}H_{14}O_2$	178.098	-5.3	151.0751, 136.0517, 91.0541	11.781	0.17	0.01
73	1-(4-hydroxy phenyl)prop ane-1,2-diol	$C_9H_{12}O_3$	168.079	-0.59	150.1272, 80.0617, 106.0408, 79.0539	12.143	0.02	/
74	kadsurin A	$C_{21}H_{24}O_6$	372.156	-4.47	285.0748, 119.0489, 123.08, 91.054, 147.0437	12.244	0.02	<0.01

75	5-hydroxy-1-tetralone	$C_{10}H_{10}O_2$	162.067	-5.13	135.044, 145.0647	12.527	0.07	/
76	16α-hydroxy estrone	$C_{18}H_{22}O_3$	286.157	-0.45	225.2224, 253.2162, 299.2575	12.791	0.08	0.02
77	α-linolenic acid	$C_{18}H_{30}O_2$	278.223	-6.41	279.231, 123.116, 109.101, 123.116	12.916	0.04	<0.01
78	9-Oxo-10(E), 12(E)-octadecadienoic acid	$C_{18}H_{30}O_3$	294.218	-6	95.0489, 277.2154	13.405	0.04	<0.01
79	ergosterol acetate	$C_{32}H_{50}O_2$	466.381	-1.07	69.068, 121.101, 309.2525	13.879	0.03	/
80	ferulic acid	$C_{10}H_{10}O_4$	194.056	-8.81	177.05428,134.036, 163.039, 149.06, 117.033	13.886	0.01	/
81	linoleoyl ethanolamide	$C_{20}H_{37}NO_2$	323.28	-8.13	324.289, 109.101, 55.0545	14.38	0.02	/
82	16-hydroxyhexadecanoic acid	$C_{16}H_{32}O_3$	272.235	-0.23	225.222, 253.217, 271.228	15.376	0.46	0.10

/, not detected.
